# Pulmonary Renal Syndrome After Streptococcal Pharyngitis: A Case Report

**DOI:** 10.1177/2324709616646127

**Published:** 2016-05-10

**Authors:** Gopi Mara-Koosham, Karl Stoltze, Jeffrey Aday, Patrick Rendon

**Affiliations:** 1 University of New Mexico, Albuquerque, NM, USA

**Keywords:** diffuse alveolar hemorrhage, post-streptococcal glomerulonephritis, pulmonary renal syndrome, plasmapheresis

## Abstract

Pulmonary renal syndrome is a class of small vessel vasculitides that are characterized by the dual presentation of diffuse alveolar hemorrhage (DAH) and glomerulonephritis. Pulmonary renal syndrome has multiple etiologies, but its development has been rarely reported following infection with group A streptococcus. We present the case of a 36-year-old Native American male who was transferred to our facility due to refractory hypoxic respiratory failure. He had been diagnosed with streptococcal pharyngitis 2 weeks prior to admission. Given the presence of hemoptysis, bronchoscopy was performed and was consistent with DAH. Urinalysis demonstrated hematuria and proteinuria, in the setting of elevated creatinine and blood urea nitrogen. Additionally, antistreptolysin O titer was positive. Given the constellation of laboratory findings and history of streptococcal pharyngitis, the patient was diagnosed with PRS secondary to streptococcal infection. High-dose methylprednisolone was initiated with concomitant plasmapheresis. He was extubated successfully after his respiratory status improved and was eventually discharged home after making a full recovery within 2 weeks after admission. This case illustrates the importance of clinically relevant sequelae of streptococcal infection as well as the appropriate treatment of PRS secondary to streptococcal pharyngitis with plasmapheresis and intravenous corticosteroids.

## Introduction

Pulmonary renal syndrome (PRS) is defined by a combination of diffuse alveolar hemorrhage (DAH) and glomerulonephritis. It involves rampant inflammation in both the lungs and the kidneys, leading to their simultaneous rapid deterioration. The majority of patients require admission to the intensive care unit, and despite optimal intervention, the mortality can reach 50%.^1^ The syndrome is predominantly autoimmune in nature, with pathogenesis resulting from autoantibodies and immune complexes, as seen in Goodpasture’s syndrome and systemic lupus erythematosus. Given the catastrophic consequences of delayed detection and treatment, timely consideration of this syndrome is paramount. The mainstay of treatment includes initiation of high-dose corticosteroids and plasmapheresis when appropriate. But the immunosuppressive nature of this treatment renders the patients susceptible to life-threatening infections. In exceedingly infrequent cases, PRS can develop *following* streptococcal infection. Herein, we present the unusual case of a patient who developed PRS 2 weeks *after* streptococcal pharyngitis.

## Case Report

A 36-year-old Native American man with history of tobacco use was transferred from an outside hospital with a hyperacute presentation of hemoptysis and rapidly worsening respiratory distress. At the time of admission to our facility, he was intubated with an oxygen saturation of 88% and a pulse of 109 beats per minute. He was obtunded and sedated on a ventilator. Physical exam revealed decreased breath sounds bilaterally and mild generalized edema in his lower extremities. The patient had oliguria (~170 mL over 24 hours). Outside medical records indicated that he was diagnosed with streptococcal pharyngitis (testing methodology not stated) 2 weeks before admission and was prescribed penicillin. He had no history of recent travel, autoimmune or clotting disorders.

Abnormal laboratory findings on admission included white blood cell count 20 800/µL, hemoglobin 12.8 g/dL, hematocrit 39%, C-reactive protein 11.5 mg/dL, fibrinogen 566 mg/dL, glucose 125 mg/dL, calcium 8 mg/dL, phosphorus 5.8 mg/dL, magnesium 2.8 mg/dL, albumin 2.5 g/dL, globulin gap 5.3 g/dL. An arterial blood gas analysis revealed the following values: pH 7.29, pO_2_ 58 mm Hg, pCO_2_ 43 mm Hg (Table 1). Creatinine was 1.78 mg/dL with a blood urea nitrogen level of 52 mg/dL. Urinalysis showed proteinuria and hematuria with multiple dysmorphic red blood cells in the urine sediment. Antistreptolysin O (ASO) antibodies were elevated at 1270 IU/mL, suggesting recent infection with group A streptococcus. C3 (9 mg/dL) and C4 (8.9 mg/dL) were also measured.

**Table 1.** Laboratory Findings of the Patient on the Day of Admission.

CBC with differential	WBC 20.8; RBC 4.35; platelets 380; Hgb 12.8; HCT 39; MCV 90; MCHC 32.6; RDWC 14.2; protime 14.6 seconds; INR 1.16; total bilirubin 0.8 (direct 0.3; indirect 0.5)
CRP	11.5 mg/dL
Albumin	2.5 g/dL
Toxicology screen	Negative for amphetamines, barbiturates, benzodiazepines, cannabinoids, cocaine, methadone, opioids, ethanol
Arterial blood gas	pH 7.29; pCO_2_ 43 mm Hg; pO_2_ 58 mm Hg; HCO_3_^−^ 20 mEq/L
Rheumatology panel	Negative for rheumatoid factor, anti-DNA, anti-neutrophil cytoplasm, anti-proteinase-3, anti-myeloperoxidase, anti-Smith Abs, anti-RNP, SS-A, SS-B, anti-GBM, cyclic citrullinated peptide
C3	9 mg/dL
C4	8.9 mg/dL
ASO	1270 IU/mL
Fibrinogen	566 mg/dL
Urine analysis	Cloudy, specific gravity 1.046; RBC >150/HPF; WBC 25/HPF; protein 100 mg/dL

Abbreviations: WBC, white blood cell; RBC, red blood cell; Hgb, hemoglobin; HCT, hematocrit; MCV, Mean corpuscular volume; MCHC, mean corpuscular hemoglobin concentration; RDWC, red blood cell distribution width; INR, international normalized ratio; CRP, C-reactive protein; ASO, anti-streptolysin O; HPF, high-power field.

**Figure 1.**
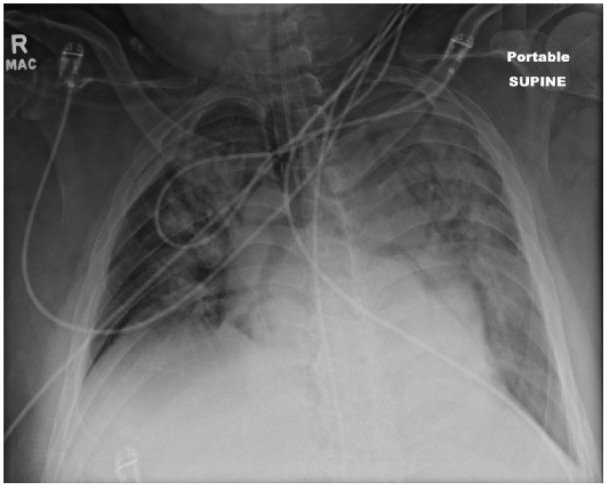
AP view of patient’s chest radiograph showing bilateral airspace consolidation and cardiomegaly suggestive of pulmonary edema.

A chest X-ray (Figure 1) noted bilateral airspace consolidation. An outside chest computed tomography scan showed diffuse centralized alveolar densities in both lungs suggestive of alveolar hemorrhage. Due to hemoptysis, a bronchoscopy was performed, which revealed a bloody lavage and erythematous mucosa in the main stem bronchi and subsegments. The bronchoalveolar lavage consisted predominantly of neutrophils with a few mononuclear cells. The culture had no growth. The patient was started on intravenous methylprednisolone (1 g/day) and plasmapheresis.

**Figure 2.**
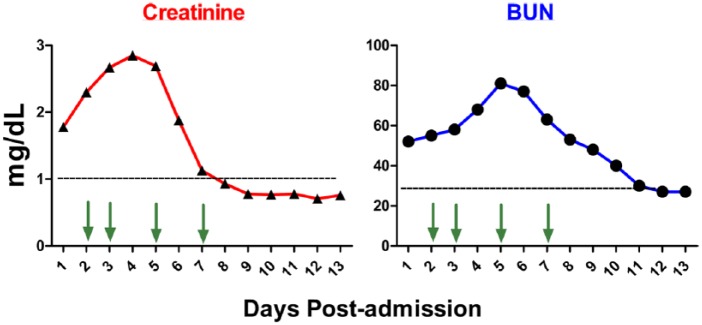
Plasmapheresis and high-dose corticosteroid treatment improved the patient’s renal function. Patient was started on daily intravenous methylprednisolone treatment (1 g/day) and subjected to plasmapheresis on days 2, 3, 5, and 7 postadmission (indicated by arrows). Creatinine and blood urea nitrogen were measured daily.

**Figure 3.**
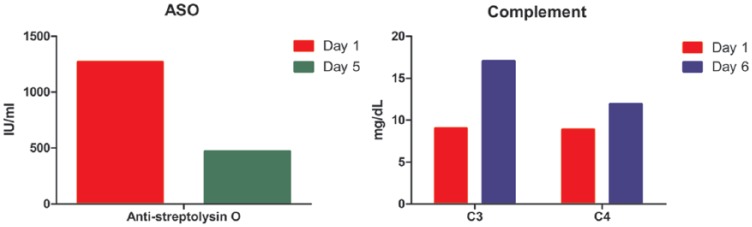
Plasmapheresis and high-dose corticosteroid treatment decreased the ASO titers while increasing the C3 and C4 levels. Patient was started on daily intravenous methylprednisolone treatment (1 g/day) and subjected to plasmapheresis on days 2, 3, 5, and 7 postadmission. Antistreptolysin O titers and C3 and C4 levels were measured on the indicated days.

The patient’s renal function worsened in the first 3 days and was restored after 4 sessions of plasmapheresis during the first week (Figure 2). The ASO levels showed progressive decline with each session of serum exchange (Figure 3). Immunoassays including antinuclear antibody, anti–double stranded DNA, antiphospholipid, antiglomerular basement, and antineutrophil cytoplasmic antibodies were negative. Infectious process was ruled out by testing including chest X-ray, urinalysis, and blood, urine, and sputum cultures. The patient was in critical condition, and in the setting of the patient’s rapid improvement with the treatment, the likelihood of acute post-streptococcal glomerulonephritis (APSGN) was high. Therefore, lung and kidney biopsies were not performed as the risks outweighed the benefits at the time. Given the patient’s history of pharyngitis, clinical presentation, laboratory analyses, and response to immunosuppressive regimen, he was diagnosed with PRS secondary to streptococcal pharyngitis.

The patient’s pulmonary function gradually improved, and he was extubated on day 8. His edema cleared and the majority of his laboratory findings normalized by 11 days postadmission. He noted poor adherence to penicillin treatment after streptococcal pharyngitis. After full recovery, he was discharged to his home on day 12 with a tapering dose of steroids.

## Discussion

This case describes a patient with a rare and unusual presentation of DAH and glomerulonephritis after a recent streptococcal infection. So far, fewer than 5 such cases have been documented worldwide.^2^ Our patient presented with the defining triad of DAH—hemoptysis, decreased hematocrit, and diffuse alveolar infiltrates. He also showed classic manifestations of APSGN including oliguric acute renal failure, microscopic hematuria, proteinuria, edema, and hypertension. Compared to previous case reports where the patients were treated with corticosteroids alone, our patient received concurrent steroids and plasmapheresis. This dual therapy may have hastened his complete recovery in 2 weeks without the need for renal biopsy as compared to 3 weeks seen in previous case reports.

Renal biopsy in previous case reports of PRS secondary to streptococcal infection showed enlarged glomeruli, neutrophilic infiltrates, IgG and C3 deposition along the mesangium—findings consistent with APSGN. Patients with a classic presentation of acute nephritic syndrome after a streptococcal infection do not generally undergo renal biopsy. Atypical features that would prompt a biopsy include rapidly progressive glomerulonephritis, persistent gross hematuria, hypertension or nephritic syndrome, extrarenal manifestations, latency period less than 10 days to onset of renal disease, hypocomplementemia lasting greater than 6 weeks, and a patient less than 2 years of age, none of which our patient exhibited.^3^ Given the compelling history of streptococcal infection and concordant laboratory values suggesting APSGN, along with rapid improvement in renal function with plasmapheresis and methylprednisolone, renal biopsy was not performed in this patient. The decision to start the patient on an immunosuppressive regimen was reached based on classic findings of DAH and impaired renal function, although 30% to 35% of patients with DAH may present without hemoptysis.^4^ Chest radiographs are normal in 22% of the patients.^5^ These unusual presentations of PRS can make the diagnosis and management potentially difficult.

The pathogenesis of PRS in relation to streptococcal infection is poorly understood. Released nephritogenic antigens including nephritis-associated plasmin receptor and streptococcal pyrogenic exotoxin B have been implicated in the development of APSGN.^5,6^ Strains that release these antigens vary regionally. They accumulate in the glomeruli, trap and maintain the activity of plasmin, and induce glomerular basement membrane degradation. The host immune reaction activates complement and produces circulating antibodies, thereby forming immune complexes. These complexes, along with neutrophils and macrophages, can infiltrate the altered glomerular basement membrane and accumulate in the subepithelial space as humps. Whether similar mode of inflammation also occurs in the lungs warrants investigation.

In conclusion, this case demonstrates APSGN as a rare, high-morbidity complication of streptococcal pharyngitis. Furthermore, it illustrates that APSGN can lead to the development of alveolar hemorrhage via PRS. Thus, in the face of a negative autoimmune workup, a thorough medical history and investigation can lead to the recognition of recent streptococcal infection as the source of PRS. An understanding of the autoimmune behavior of streptococcal infection is vital in the management of these patients. This facilitates timely initiation of plasmapheresis and high-dose corticosteroids, for which evidence supports their use in resolving this syndrome.^1,6^ Therefore, to improve the mortality of these patients it is essential for clinicians to be mindful of the rare, yet significant complications of common conditions such as streptococcal pharyngitis.^1^
